# Epidemiology of COVID-19 in Prisons, England, 2020

**DOI:** 10.3201/eid2708.204920

**Published:** 2021-08

**Authors:** Wendy M. Rice, Dimple Y. Chudasama, James Lewis, Francis Senyah, Isaac Florence, Simon Thelwall, Lisa Glaser, Maciej Czachorowski, Emma Plugge, Hilary Kirkbride, Gavin Dabrera, Theresa Lamagni

**Affiliations:** Public Health England, London, UK

**Keywords:** prisons, incarcerated persons, outbreaks, COVID-19, England, SARS-CoV-2, severe acute respiratory syndrome coronavirus 2, viruses, respiratory infections, zoonoses, vaccines, coronavirus disease, England, United Kingdom

## Abstract

Using laboratory data and a novel address matching methodology, we identified 734 cases of coronavirus disease in 88 prisons in England during March 16–October 12, 2020. An additional 412 cases were identified in prison staff and household members. We identified 84 prison outbreaks involving 86% of all prison-associated cases.

Incarcerated persons are at an increased risk for severe acute respiratory syndrome coronavirus 2 (SARS-CoV-2) transmission and illness because of both the prison environment and the vulnerability of the residents (*1*,*2*). To limit spread in prisons in England, visitation restrictions were introduced, the population was compartmentalized to limit movement, and an early release scheme was put in place (*3*,*4*). As in the general population, only those admitted to a hospital were tested for SARS-CoV-2 initially, but testing was expanded to all symptomatic cases in late May 2020, specifically persons with cough, fever, or anosmia.

Outbreaks of coronavirus disease (COVID-19) have been reported in correctional facilities (*5*–*8*). We describe characteristics and outcomes for prison-associated COVID-19 cases in England reported to Public Health England (PHE) in March 16–October 12, 2020.

## The Study

COVID-19 cases confirmed by real-time PCR in England must be reported to PHE’s laboratory reporting system (Second Generation Surveillance System [SGSS]) in accordance with statutory legislation (*9*). Prison residence was identified from case addresses reported by laboratories or the NHS database–registered address (*9*).

We used a previously described process to match case data against a national database of properties (AddressBase Premium; Ordnance Survey, https://www.ordnancesurvey.co.uk) listed by Unique Property Reference Number (UPRN) (*2*). We identified prisons using the property classes designated by UPRN. We used ESRI LocatorHub software (https://www.esriuk.com) for exact matching of case addresses to AddressBase. We used fuzzy matching on failed records and manually matched remaining records.

**Figure 1:**
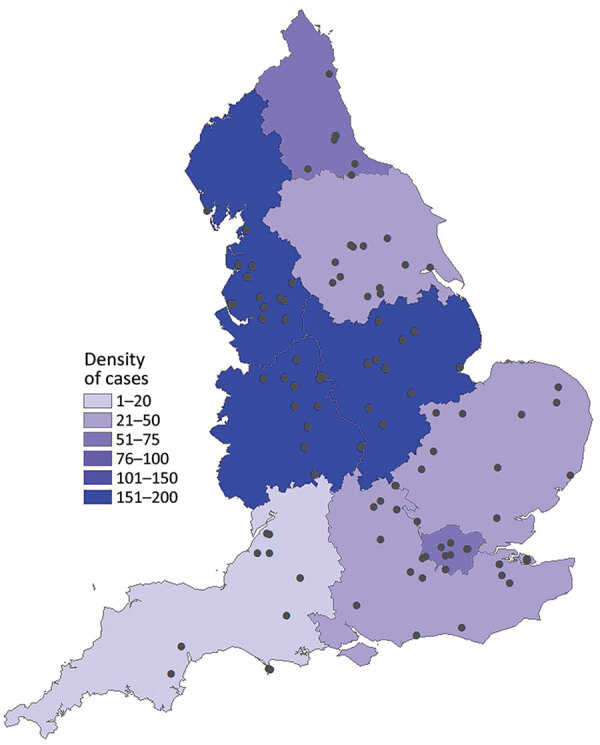
Cases of coronavirus disease in incarcerated persons, England, March 16–October 12, 2020. Dots indicate prison locations. Shading indicates density of cases by Public Health England center.

Laboratory records from national key worker testing were the sources for identifying prison staff and of symptomatic household members of key workers also eligible for testing. We were not able to link prison staff–associated cases to specific facilities because workers’ residential addresses and not workplace addresses were provided; we could not extrapolate workplace on the basis of residence given the regional prison distribution (Figure 1).

We defined associated deaths as deaths in cases occurring <60 days from first positive specimen date or in cases for which COVID-19 was on the death certificate. We calculated incidence in incarcerated persons using official prison denominators for September 2020 (*10*).

We defined outbreaks in prisons as >2 cases within 14 days (by specimen date) residing at the same location (UPRN). We extracted records for cases identified March 16–October 12, 2020, and analyzed them using Stata version 15 (StataCorp, https://www.stata.com). The first laboratory-confirmed COVID-19 case in an incarcerated person in England was identified on March 16, 2020, in a high-security prison. We identified 734 incarcerated case-patients, accounting for 0.14% of all cases reported through October 12, 2020 in England (N = 527,225); we also identified 412 cases in prison staff and their households.

**Figure 2:**
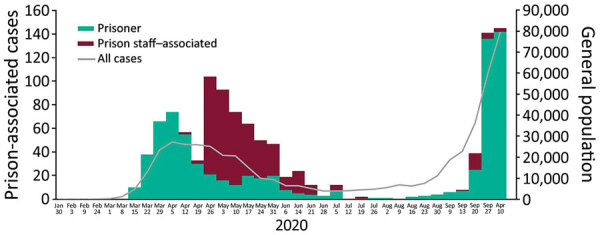
Laboratory-confirmed coronavirus disease cases associated with prisons in England (incarcerated persons, prison staff, and families) by testing date compared to all cases in England, March 16–October 12, 2020. Scales for the y-axes differ substantially to underscore patterns but do not permit direct comparisons.

Most (52%, 380/734) incarcerated cases were reported before June 6; a second wave was reported in mid-September (Figure 1). The crude incidence in incarcerated persons in England was 988.1/100,000 population, which was not significantly different than incidence in the general population, at 935.3/100,000 population (relative risk 1.05; p = 0.14). Incidence rates varied between prisons, from 0 to 14,171.4/100,000 population. Of the 112 prisons in England, 88 (78.6%) were identified as having >1 confirmed case (Figure 1). Most prison staff–associated cases were identified after the introduction of key worker testing in April 2020, a total of 303 (74%) staff cases during April 28–May 31, mirroring the trend in England (Figure 2).

Ethnicity data were available for 652 incarcerated cases. Of those, 74.3% (n = 507) identified as White, 7.9% (n = 54) Asian, and 6.4% (n = 44) Black, compared with the general population that was 86% White, 7.5% Asian, and 3.3% Black (*11*). Most staff-associated case-patients identified as White (86.6%, 367/412) (Table).

**Table:** Demographic characteristics of COVID-19 cases in incarcerated persons and in persons associated with prison staff, England, March 16–October 12, 2020 *

Characteristic	Incarcerated persons	Prison staff–associated	No. deaths	CFR† (95% CI)
Total, N = 1,157	734 (63.5%)	412 (35.6%)	23 (1.99%)	3.13 (2.00–4.67)
Age group, y				
0–17	5	14	0	0.00‡
18–21	31	12	0	0.00
22–45	435	223	4	0.92 (0.02–2.34)
46–65	192	153	8	4.17 (1.82–8.04)
>66	71	10	11	15.5 (8.0–26.03)
Sex				
F	46	166	2	4.35 (0.05–14.84)
M	688	242	22	3.05 (1.9–4.63)
Unknown	0	4	0	0.00
Race/ethnicity‡				
White/White British	507	318	20	3.94 (2.43–6.03)
Asian/Asian British	54	33	NA	‡
Black/Black British	44	9	NA	‡
Mixed	20	4	NA	0.00
Other	27	3	NA	0.00
Unknown	82	45	NA	0.00
Prison type				
Category C (trainer)	259	NA	7	2.70 (1.09–5.49)
Local	193	NA	8	4.15 (1.81–8.0)
Category B (high security, trainer)	138	NA	2	1.45 (0.18–5.14)
Female	43	NA	2	4.65 (0.57–15.81)
Category A (maximum security)	40	NA	2	5.00 (0.61–24.29)
Open	27	NA	2	7.41 (0.91–24.29)
Youth detention	15	NA	0	0.00

*No deaths were reported among prison staff or their associated cases. CFR, case-fatality rate; COVID-19, coronavirus disease; NA, not available. †CFR calculated using only incarcerated case data. ‡CFR and 95% CIs for non-White groups not included due to small numbers reported in other categories (<5 cases).

Twenty-three COVID-19-related deaths were identified, giving a case-fatality ratio (CFR) of 3.13%; the CFR for England was 8.0% over the study period. The CFR was highest for those reported to be White (3.94%, 20/507); differences by ethnicity were not statistically significant (p = 0.32) (Table).

The number of cases at each prison was 1–124 (IQR 3–7); the upper range resulted from a single outbreak. Overall, 87% of incarcerated cases (n = 638) were associated with an outbreak; we identified a total of 84 prison outbreaks. Eighteen deaths occurred across all outbreaks. For staff-associated cases, clustering by time and place was seen within the same household for 73 cases, including 14 children >18 of age.

## Conclusions

In this study, we aimed to use routine laboratory surveillance data to describe the first wave of COVID-19 cases associated with prisons in England. Because nearly half the prisons in England are overcrowded (*12*), cases in these environments require monitoring and prompt response. Nearly all cases in incarcerated persons were associated with an outbreak. Future work should examine the value of genome sequencing to link outbreak cases molecularly.

Although we saw no difference in the crude incidence rates between incarcerated persons and the general population, infection rates were likely underestimated because asymptomatic persons were not tested. In prisons, testing of asymptomatic persons could be employed at the discretion of local government but was usually only done in larger or more severe outbreaks. Other studies have demonstrated asymptomatic detections during outbreaks in other institutional settings (*13*); wider asymptomatic testing was not introduced in England until 2021.

Testing hesitancy has been reported elsewhere (*6*), which could also affect the crude rates we report. Inaccurate address information for incarcerated case-patients could also lead to underestimation. Trends in deaths among the incarcerated differs from reports elsewhere (*7*). Calculating and comparing CFRs in subsequent waves in these facilities could help to understand this trend.

Sixteen percent of case-patients were from a Black, Asian, or other minority ethnic background, despite making up over a quarter of the prison population and 13% of the general population (*12*) (Table). The differences in infection rates observed by ethnicity may relate to age-related conditions in this population. We noted a higher proportion of White incarcerated cases >65 years of age; increased age is a known risk factor for severe COVID-19 infection (*12*,*14*). Older age groups also experience a high burden of noncommunicable diseases, putting them at increased risk for more severe infection (*14*). Possible differences in acceptance of testing by age or ethnicity should also be considered relating to these different rates.

The inability to distinguish key workers from household members using these data limited our ability to determine household transmission direction but indicates spread. We were unable to assess the potential role of prison staff–associated cases in seeding prison outbreaks on the basis of routine laboratory data. Other studies have indicated that cases associated with correctional facilities can contribute to additional spread in local communities (*15*), supporting the potential benefit of routine screening of staff to prevent seeding of COVID-19.

Despite limitations, this study adds to the growing evidence base addressing the impact of COVID-19 in prisons. We demonstrate the utility of a highly sensitive address matching methodology to help enhance COVID-19 surveillance. Prison-associated cases make up <1% of COVID-19 cases in England. Because of the increased risk for rapid spread in these environments and the effects of outbreak management on the health of the incarcerated population, being able to identify early signals of increasing case numbers is of great importance for protecting these vulnerable groups.
